# Current Knowledge on Factor V Leiden Mutation as a Risk Factor for Recurrent Venous Thromboembolism: A Systematic Review and Meta-Analysis

**DOI:** 10.3389/fcvm.2022.883986

**Published:** 2022-04-07

**Authors:** Daria Eppenberger, Henning Nilius, Betsy Anagnostelis, Carola A. Huber, Michael Nagler

**Affiliations:** ^1^Department of Clinical Chemistry, Inselspital, Bern University Hospital, University of Bern, Bern, Switzerland; ^2^Medical Library Research Support Service, University Library of Bern, University of Bern, Bern, Switzerland; ^3^Department of Health Sciences, Helsana Insurance Group, Zürich, Switzerland

**Keywords:** heterozygous factor V Leiden mutation, recurrent venous thromboembolism, prospective cohort studies, systematic review, risk factors

## Abstract

**Background:**

Thrombophilia screening is widely done in clinical practice, and it is claimed that the extent of venous thromboembolism (VTE) recurrence risk in patients with common defects is still not fully understood.

**Aim:**

We aimed to summarize data of all observational studies prospectively assessing the association of heterozygous factor V Leiden (FVL) mutation and recurrent VTE in patients with VTE, and to calculate pooled relative risks (RR), overall and in various subgroups.

**Methods:**

We searched MEDLINE and EMBASE databases for cohort studies prospectively assessing VTE recurrence in patients with and without FVL mutation (PROSPERO: CRD42021182800). Data were extracted on cohort and study-level. The methodological quality was assessed using the Newcastle-Ottawa Scale (NOS). RR were calculated overall and in subgroups using a random-effects model.

**Results:**

From 31 cohorts, 24 studies were finally included summarizing 13,571 patients. Heterozygous FVL mutation was identified in 2,840 individuals (21%). The methodological quality was estimated to be high in 20 studies (83%). The overall RR was 1.46 (95% CI: 1.31, 1.64), consistent across subgroups.

**Conclusions:**

Pooling all high-quality epidemiological data, the risk of recurrent VTE was increased by 46% in patients with heterozygous FVL mutation. Against the background of established risk factors, the FVL mutation plays only a marginal role in the risk assessment for recurrent VTE.

## Introduction

Thrombophilia screening is still a popular tool in the workup of patients with venous thromboembolism (VTE) (1, 2). VTE is one of the most common cardiovascular diseases associated with high morbidity and mortality (3–7). More than 25% of unselected patients experience recurrent events, potentially resulting in a reduced quality of life or even death (8, 9). Thus, preventing recurrent VTE is an important goal of secondary prevention (4, 10–12). To accomplish this, high-risk patients must be identified (9, 13). Given the clustering of VTE in families or even in individuals, genetic factors are considered as promising targets (14–16). The most common inherited thrombophilia is heterozygous factor V Leiden (FVL) mutation, which is acknowledged as a relevant risk factor for first VTE (17, 18). Earlier investigations suggest a moderately increased risk only and current guidelines do not suggest thrombophilia testing in unselected patients (1, 19–26). However, the selection criteria are largely unclear and thrombophilia screening (including FVL mutation) is still frequently done in clinical practice (1, 2, 9, 20, 27–33). Besides, some authors claim that the knowledge is still limited, particularly within subgroups of patients, and that the presence of FVL mutation might sum up with other risk factors resulting in a modified treatment recommendation (14, 34, 35).

Various previous studies observed the association between the presence of FVL mutation and the risk of VTE recurrence and the results are conflicting. Some studies concluded that heterozygous FVL mutation increases the risk (10, 12, 36–41) and others do not (38, 42–48). In particular, some authors raise the question of whether FVL mutation increases the recurrence risk in specific subgroups such as men (36), young women without hormonal treatment (38), or cancer patients (49, 50). Indeed, FVL mutation was also detected in various genetic profiling studies (10, 23, 40, 41, 51–54), and it was included in one clinical prediction model (53). Thus, whether or not FVL mutation increases the risk of recurrent VTE to a relevant degree is not fully understood, and more data are needed to clarify this issue.

### Aim

In a systematic review and meta-analysis, we aimed to summarize data of all observational studies prospectively assessing the association of heterozygous FVL mutation and recurrent VTE. We aimed to calculate relative risks (RR) overall and in various subgroups of patients. To set this into context, we observed the frequency of testing in Switzerland using a large claim-based dataset.

## Methods

The study protocol was submitted to the PROSPERO international prospective register of systematic reviews (#CRD42021182800) and the manuscript was written according to the Preferred Reporting Items for Systematic Reviews and Meta-Analysis (PRISMA) (55).

### Search Strategy, Screening, and Identification

A comprehensive search strategy for MEDLINE (1946 to February 03, 2022) and EMBASE (1974 to 2022 February 03) databases was developed, and the Ovid interface used (Supplementary List 1). The search strategy was based on three elements: heterozygous FVL mutation (patients); recurrent VTE (outcome); and prospective cohort study (study design). The search strategy was improved using keywords found in key publications and no limits were applied. The sensitivity was tested in eight key publications (100%). The literature search was completed by hand search using reference lists of articles retrieved. All included studies were checked for published errata. The last search run was done on the fourth of February 2022. All records were carefully assessed for eligibility by screening of title, abstract and full text by two reviewers in duplicate (D.E., M.N.).

### Study Eligibility

The following inclusion criteria were applied: (a) prospective cohort studies, (b) patients tested for FVL mutation/ activated protein C resistance (APCR) at baseline, (c) objectively confirmed VTE, (d) recurrent VTE defined as primary outcome, and (e) numbers of recurrences or recurrence rates reported separately in patients with and without FVL mutation. Exclusion criteria were (1) retrospective studies, (2) case-control studies, case reports, and (3) studies conducted in close subgroups (e.g., children, perioperative VTE, upper extremity deep venous thrombosis, and homozygous FVL mutation). Articles based on the same cohort were compiled and the publication with the (a) highest number of patients, and (b) most complete clinical data were selected for meta-analysis.

### Definition of Outcomes

Recurrent VTE was defined as objectively confirmed VTE. For deep venous thrombosis (DVT), one of the following imaging techniques must have been used: venography, duplex sonography, or compression ultrasonography. For pulmonary embolism (PE), ventilation-perfusion scan, spiral computed tomography, or pulmonary angiography should have been used (56–58).

### Data Extraction

First, several characteristics were retrieved to summarize each cohort: name of cohort, country, setting (type of health care institution), time period of patient recruitment, inclusion criteria and all publications. Secondly, detailed data were extracted out of the selected publication for meta-analysis: first author, year of publication, age of patients (mean or median), total number of patients, number of female patients, number of FVL mutation patients (at baseline), location of initial VTE (isolated distal DVT, proximal DVT/PE or mixed DVT/PE), triggering factor first VTE (unprovoked, provoked, mixed), duration of anticoagulation (months), type of anticoagulation [Vitamin-K antagonist (VKA), direct oral anticoagulants (DOAC)], absolute number of patients with unprovoked VTE, number of cancer patients, observation period (months), total number of patients with recurrence, number of FVL mutation patients with recurrence, number of non-FVL mutation patients with recurrence and recurrence rate of FVL mutation patients.

### Assessment of Methodological Quality

The methodological quality of the primary studies was assessed using the Newcastle-Ottawa Scale (NOS) for cohort studies (59). The following three domains were applied: (a) selection of patients, (b) comparability of study groups, and (c) outcome of interest. The questions were modified to fit the present research question (Supplementary List 2). The assessment was done in duplicate (D.E., M.N.) and discrepancies were resolved by discussion.

### Frequency of FVL Testing

To set this analysis into context, we assessed the frequency and trends of testing for FVL mutation in the Swiss health care system. Health care claims data of Helsana, one of the largest Swiss health insurance companies were used. Approximately 15% of the Swiss population are insured with Helsana for obligatory basic insurance, and the population is considered representative (60, 61). All invoices submitted for reimbursement for FVL mutation (#6200.64) and APCR (#1086.00) of the list of analyses from the Federal Office of Public Health were retrieved between 2014 and 2020 (62).

### Statistical Analysis

Using the extracted data, the relative risks (RR) and their 95% confidence intervals (CI) were calculated for each primary study. The RRs were then calculated using a random-effects model based on the Mantel-Haenszel estimator, and the corresponding 95% CI were computed. Heterogeneity between studies was assessed using Higgins' *I*^2^. All analyses were performed using the “meta,” “etaphor,” and “dmetar” packages for R. As a first sensitivity analysis, a leave-one-out analysis was performed to check for outliers. Studentized residuals and Cook's distance were calculated, and studies with studentized residuals outside of −1 and 1, and Cook's distances >50% of the lower tail of a Chi-square distribution with *p* (*p* = number of model coefficients) degrees of freedom were flagged as potentially influential outliers. These studies were excluded from the overall analysis. Furthermore, subgroup analysis was performed for the following subgroups: Year of publication (<2000, 2000–2010, >2010), location of the initial VTE (mixed, proximal DVT/PE), presence of triggering risk factors for the initial VTE (unprovoked, provoked, and mixed), the anticoagulation drug used (VKA, DOAC), and the presence of cancer (no cancer, mixed). A funnel plot was additionally created to assess publication bias.

## Results

### Cohort and Study Identification and Selection

**Figure 1:**
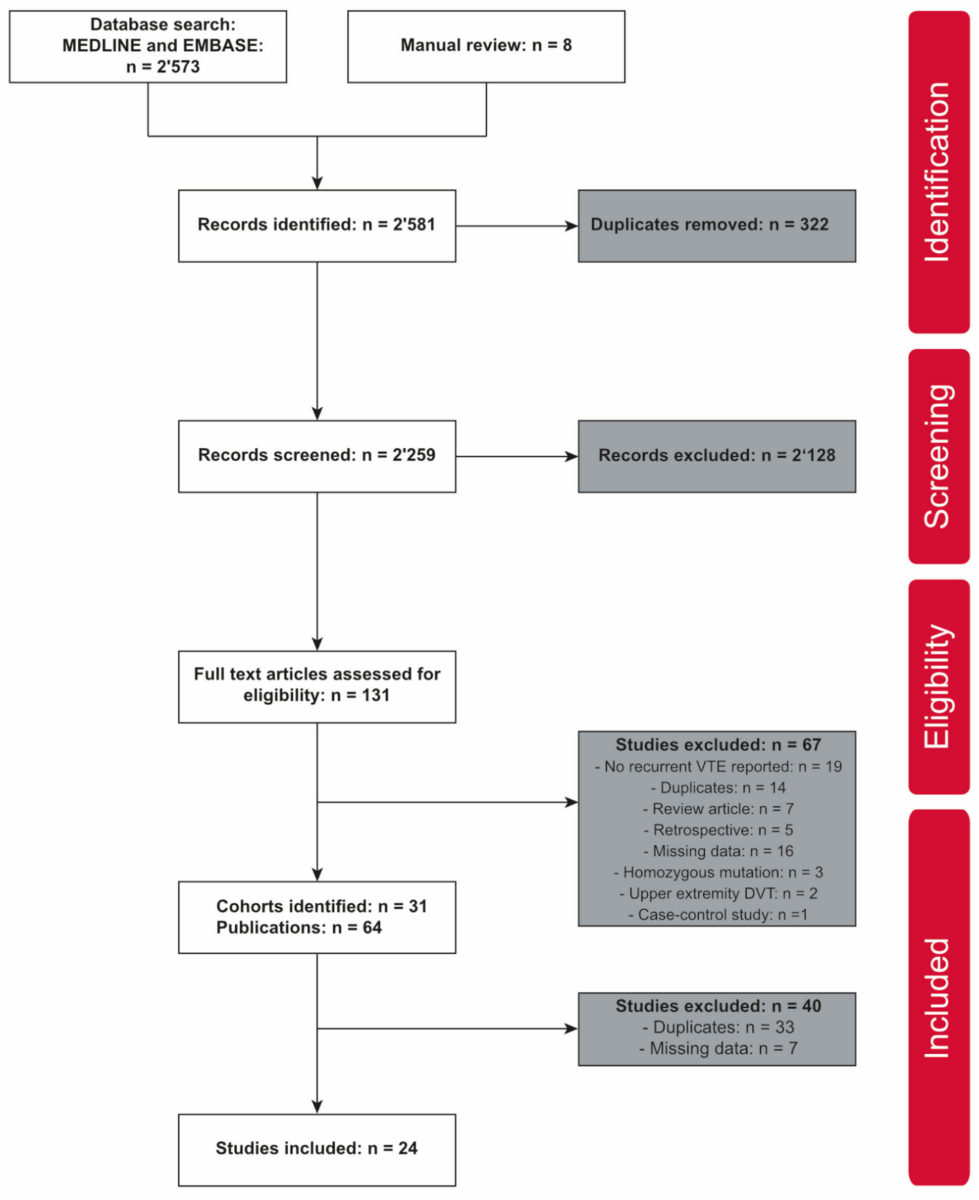
PRISMA flowchart.

The literature search retrieved a total of 2,581 publications, 2,573 accessed in MEDLINE and EMBASE databases, and eight identified by manual review (Figure 1). After removing duplicates, the title and abstract of the 2,259 remaining publications were screened, giving 131 publications for full-text screening (including 100 journal articles and 21 conference abstracts). A total of 67 publications were excluded with reasons. Eventually, 31 different prospective cohort studies were identified (Table 1; Figure 1). Per cohort, the publication with the most complete clinical data was selected for further analysis. No publication with sufficient data were identified in seven cohorts (44, 45, 47, 54, 80, 101, 104). Twenty-four publications were finally considered for meta-analysis (Figure 1) (17, 36–41, 46, 48, 63–65, 67, 69–71, 73, 75, 78, 82–84, 86, 103).

**Table 1:** Characteristics of prospective cohort studies including patients with VTE.

**Name of cohort**	**Time period of patient recruitment**	**Country**	**Setting**	**Inclusion criteria**	**Identified publications**
PHS: Physicians' Health study	1982 to 1983	USA	Male physicians residing in the United States	VTE; U.S. male physicians 40–84 years	(63)
DURAC trial: Duration of Anticoagulation study	April 1988 to April 1991	Sweden	16 secondary/ tertiary hospitals, Department of internal Medicine	First DVT/PE; age > 15 and <71	(64)
LETS: Leiden Thrombophilia Study	January 1988 to December 1992	Netherlands	3 anticoagulation clinics	First DVT including arm thrombosis; age <70	(52, 65, 66)
Padua^*^	January 1986 to June 1994	Italy	Thrombosis unit of the University of Padua	First DVT	(67, 68)
Extended anticoagulation trial	October 1994 to April 1997	Canada, USA	13 secondary/tertiary hospitals	First unprovoked proximal; DVT/PE; received OAC ≥ 3 months	(69)
EPCOT: European Prospective Cohort on Thrombophilia study	March 1994 to September 1997	Spain, Italy, Germany, UK, Netherlands, Sweden, France, Austria	9 anticoagulation clinics	In this subcohort: First DVT/PE before study entry	(70)
LIST: The Linköping Study on Thrombosis	February 1998 to January 2000	Sweden	Linköping University Hospital (emergency department)	VTE; age ≥ 18	(71, 72)
THRIVE III: Ximelagatran in VTE	November 1999 to October 2000	18 countries: Europe, Argentina, Brazil, Canada, Israel, Mexico, South Africa	142 secondary/tertiary hospitals	DVT/PE; age ≥ 18; received OAC for 6 months without recurrence	(17)
ELATE: The Extended Low-intensity Anticoagulation for unprovoked Thrombo-embolism	December 1998 to May 2001	Canada, USA	16 secondary/tertiary hospitals	Unprovoked proximal DVT/PE; received OAC ≥ 3 months; warfarin therapy during follow-up	(73)
CVTE: The Cambridge Venous Thromboembolism Study	August 1997 to January 2002	United Kingdom	Addenbrooke's Hospital Cambridge (thrombosis center)	First DVT/PE	(44, 52, 74)
Bologna^*^	February 1995 to February 2002	Italy	S. Orsola-Malpighi University Hospital Bologna (thrombosis center)	First DVT/PE; received OAC ≥ 3 months	(75–77)
Salamanca^*^	June 1997 to June 2002	Spain	Thrombosis and Hemostasis Section of the University Hospital of Salamanca	First DVT/PE	(46)
PORtromb project: Oporto thrombophilia study	October 1997 to November 2002	Portugal	Sao Joao University hospital (outpatients unit)	First DVT including arm thrombosis; age <40	(45)
PREVENT: Prevention of Recurrent Venous Thromboembolism trial	July 1998 to December 2002	USA, Canada, Switzerland	52 secondary/tertiary hospitals	Documented unprovoked VTE; age ≥ 30; received OAC ≥ 3 month	(47)
Italy1^*^	May 1991 to April 2003	Italy	Emergency departments of 3 secondary/tertiary hospitals	First proximal DVT/PE; received OAC 3–6 months without recurrence	(78, 79)
Italiy2^*^, AESOPUS investigators	January 1999 to July 2003	Italy	9 university or hospital centers in Italy	First proximal DVT; age ≥ 18; received OAC 3 months without recurrence	(80)
MEGA follow-up study: Multiple Environmental and Genetic Assessment of risk factors for venous thrombosis	March 1999 to September 2004	Netherlands	6 anticoagulation clinics	First DVT/PE; age <70	(53, 54, 81)
Florence	January 1999 to January 2007	Italy	Thrombosis center at University hospital Careggi Florence	First VTE	(82)
Jordan^*^	January 2005 to December 2007	Jordan	Jordan University Hospital	Acute PE	(83)
REVERSE I	2001 to 2007	Canada, France, Switzerland, USA	12 tertiary care centers	First unprovoked proximal DVT/PE; age ≥ 18; received OAC 5–7 month without recurrence	(84, 85)
AUREC: Austrian Study on Recurrent Venous Thromboembolism	July 1992 to August 2008	Austria	4 thrombosis centers in Vienna; secondary care/tertiary care	First unprovoked DVT/PE; age ≥ 18; received OAC ≥ 3 months	(11, 16, 42, 43, 86–96)
MATS: Malmö Thrombophilia Study	March 1998 to December 2008	Sweden	Skane University Hospital (emergency department)	VTE; age ≥ 18	(10, 14, 37, 97–100)
TEHS-follow up study: Thromboembolism Hormone Study	2003 to 2009	Sweden	43 secondary/tertiary hospitals	First DVT/PE; age > 18 and <64	(39)
FARIVE study: Facteurs de risqué et de récidives de la maladie thromboembolique veineuse	2003 to 2009	France	11 centers	First unprovoked DVT/PE; age ≥ 18	(12, 36)
MAISTHRO: Main-Isar-Thrombosis registry	March 2000 to February 2010	Germany	University hospital's outpatient department, Goethe University Hospital Frankfurt/Main	Acute or documented history of DVT/PE; age ≥ 18	(38)
France^*^	January 1992 to June 2011	France	Brest University Hospital	First DVT/PE; age ≥ 18 and <50; Women	(101, 102)
Madrid^*^	March 2004 to August 2013	Spain	2 University hospitals in Madrid	First unprovoked DVT/PE; age ≥ 18; received OAC ≥ 3 months	(103)
SWITCO65+: Swiss Venous Thromboembolism Cohort	September 2009 to December 2013	Switzerland	9 tertiary hospitals in Switzerland	First unprovoked DVT/PE; age ≥ 65	(48)
Germany^*^	December 2008 to December 2018	Germany	Multicenter	First VTE; age adolescents to 60 years	(40)
Egypt^*^	January 2015 to December 2020	Egypt^*^	Tanta University Hospital	First VTE; age ≥ 18	(41)
Conference Abstract I^*^	–	France	–	First proximal DVT/PE	(104)

* *No cohort name available*.

### Cohort Characteristics

Thirty-one prospective cohort studies conducted in Europe (*n* = 23), North America (*n* = 3), Europe and North America (*n* = 2), and other areas (*n* = 3) were identified. The number of publications per cohort ranged from 1 (17, 38–41, 45–48, 54, 63, 64, 69, 70, 73, 80, 82, 83, 103, 104) to 15 (86). Twenty-three cohorts included patients with a first VTE (36, 39–41, 44–46, 48, 54, 64, 65, 67, 69, 70, 75, 78, 80, 82, 84, 86, 101, 103, 104), and eight cohorts included patients with any VTE (17, 37, 38, 47, 63, 71, 73, 83). Detailed cohort characteristics are reported in Table 1.

### Studies Characteristics and Patients

Details of the primary studies included in the meta-analysis are reported in Table 2, summarizing data of 13,571 patients, including 2,840 patients with FVL mutation (21%). The number of patients varied between 72 (83) and 1,267 (37). The prevalence of FVL mutation ranged between 8.4% (36) and 28% (86). The mean or median age varied between 37 years (40) and 76 years (48). The observation periods varied from six (83) to 88 (63, 65) months. VKA were used in most studies (36, 37, 41, 46, 48, 64, 65, 67, 69, 71, 73, 75, 78, 82, 84, 86, 103), summarizing 8,654 patients (64%). DOAC were used as anticoagulant in one study (17), and the type of anticoagulant was not specified in six studies (38–40, 63, 70, 83). The inclusion criteria and the type and location of the primary event is reported in Table 1. Eight studies included patients with a first unprovoked VTE only (36, 41, 48, 69, 73, 84, 86, 103) and one study provided separate data (provoked/unprovoked) (67). Both provoked and unprovoked VTE were included in 15 primary studies (17, 37–40, 46, 63–65, 70, 71, 75, 78, 82, 83). Patients with cancer were excluded in 16 studies (36, 39–41, 46, 48, 64, 65, 67, 69, 73, 78, 82, 84, 86, 103) and not reported in two studies (63, 83). Overall, 341 cancer patients were reported in six studies (17, 37, 38, 70, 71, 75). A funnel plot is given in Supplementary Figure S1.

**Table 2:** Characteristics of studies included in meta-analysis.

**Author/year**	**Age**	**Anticoagulant used**	**Patients, total**	**Patients, FVL mutation**	**Patients with unprovoked VTE**	**Observation period**	**Recurrences, total**	**Recurrences, FVL mutation**
	**Years (mean or median)**		**Numbers**	**Numbers**	**Numbers**	**Months (mean/median)**	**Numbers (%)**	**Numbers (%)**
Simioni et al. (67)^+^ (provoked VTE)	63	VKA	106	13	0	47	10 (9.4)	4 (30.1)
Simioni et al. (67)^+^ (unprovoked VTE)	63	VKA	145	28	145	47	39 (26.9)	10 (35.7)
Kearon et al. (69)^#^ (placebo group)	58	VKA	83	19	83	9	17 (20.5)	2 (10.5)
Kearon et al. (69)^#^ (intervention group)	59	VKA	79	15	79	12	1 (1.7)	0 (0.0)
Lindmarker et al. (64)	58	VKA	467	118	267	48	65 (13.9)	19 (16.1)
Miles et al. (63)	40–84^$^	–	218	26	101	88	29 (13.3)	5 (19.2)
Palareti et al. (75)	67	VKA	599	68	282	17	58 (9.7)	15 (22.1)
Christiansen et al. (65)	45	VKA	474	84	259	88	90 (19)	19 (22.6)
Vossen et al. (70)	40	–	304	76	167	67	51 (16.8)	12 (15.8)
Wahlander et al. (17)^#^ (placebo group)	58	VKA	531	121	–	18	57 (10.7)	16 (13.2)
Wahlander et al. (17)^#^ (intervention group)	56	DOAC	549	100	–	18	9 (1.6)	2 (2)
Gonzalez-Porras et al. (46)	47	VKA	181	29	117	56	27 (14.9)	5 (17.2)
Prandoni et al. (78)	66	VKA	953	111	–	50	208 (21.8)	38 (34.2)
Poli et al. (82)	64	VKA	169	22	107	30	27 (15.9)	5 (22.7)
Eichinger et al. (86)	49	VKA	1,107	307	1,107	44	168 (15.2)	60 (19.5)
Rodger et al. (84)	53	VKA	646	100	646	18	91 (14.1)	19 (19)
Kearon et al. (73)	57	VKA	661	161	661	28	14 (2.1)	3 (1.9)
Chaireti et al. (71)	61	VKA	158	39	–	84	42 (26.5)	17 (43.6)
Obeidat et al. (83)	50	–	72	17	23	6	7 (9.7)	2 (11.8)
Sveinsdottir et al. (37)	63	VKA	1,267	339	511	58	131 (10.3)	49 (14.5)
Olie et al. (36)	49	VKA	583	49	583	27	74 (12.6)	9 (18.4)
Weingarz et al. (38)	43	–	1,221	287	299	77	261 (21.4)	63 (22)
Franco Moreno et al. (103)	61	VKA	398	106	398	21	65 (16.3)	45 (42.5)
Bruzelius et al. (39)	46	–	1,010	238	367	60	101 (10)	33 (13.9)
Mean et al. (48)	76	VKA	354	32	354	30	54 (15.3)	4 (12.5)
Limperger et al. (40)	37	–	1,012	275	223	51	178 (17.6)	68 (24.7)
Hodeib et al. (41)	52	VKA	224	60	224	50	58 (25.9)	22 (36.7)

+ *Provoked and unprovoked VTE patients were reported separately*;

# *intervention and placebo group of a randomized controlled trial were reported separately; –data not reported*;

$ *range*.

### Methodological Quality

**Figure 2:**
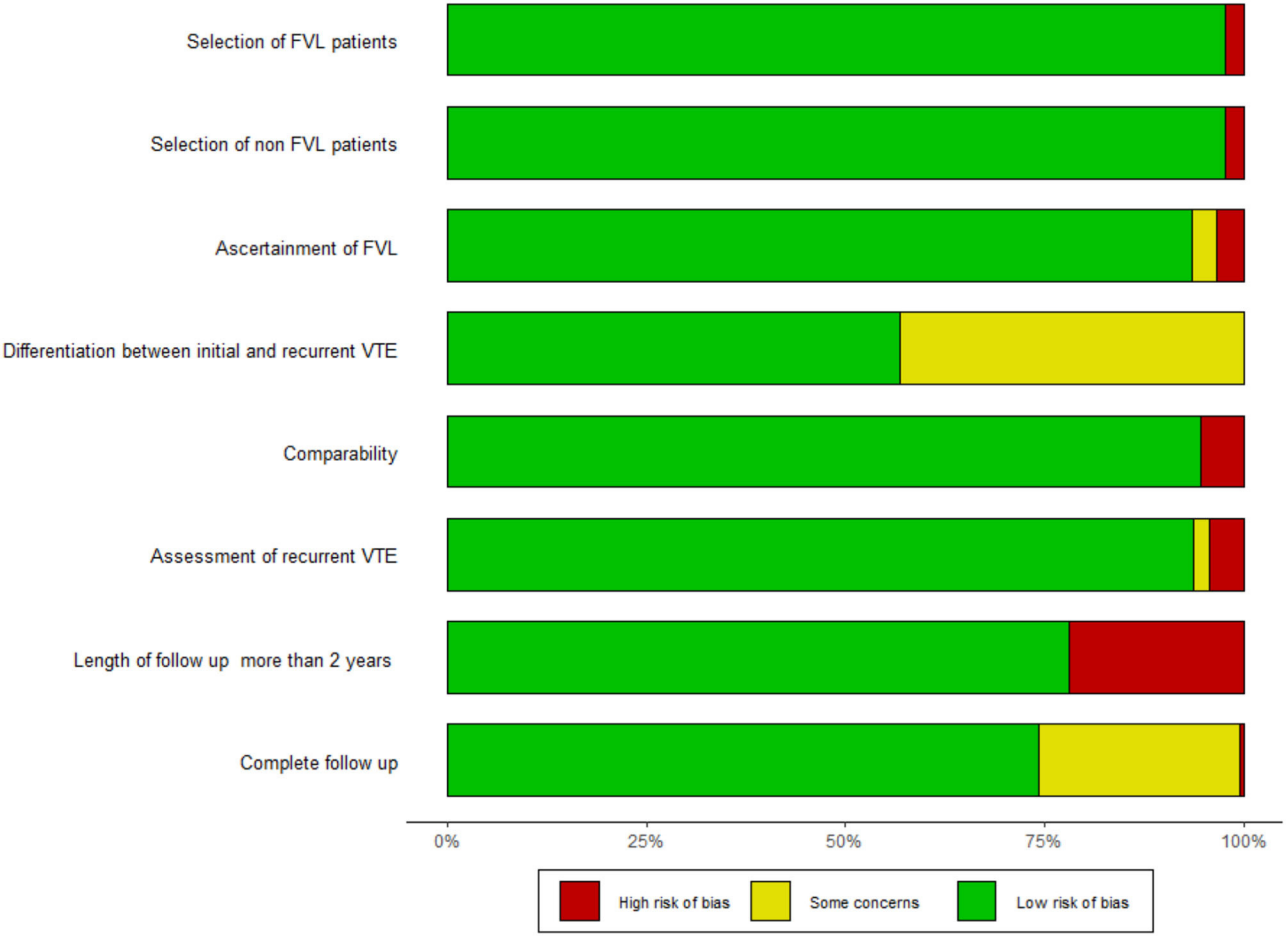
Summary of methodological quality. Rating according to the NOS questionnaire. The detailed questionnaire is shown in the Supplementary List 2.

A summary of the methodological quality according to the NOS tool is given in Figure 2; detailed results for all studies are reported in the Supplementary Table S1. With at least six NOS criteria fulfilled in twenty studies, the overall methodological quality was high (17, 36–41, 48, 63–65, 67, 69, 71, 73, 75, 78, 82, 84, 86). Three to five criteria were fulfilled by four studies (46, 70, 83, 103). The three domains most frequently not met were (1) method reported for distinguishing the initial and recurrent VTE (37, 39–41, 46, 48, 63, 65, 67, 70, 71, 83, 103), (2) follow-up longer than 2 years (17, 69, 75, 83, 84, 103), and (3) follow-up rate ≥90% of patients (17, 40, 41, 46, 48, 67, 83, 103).

### Risk of Recurrent VTE Among FVL Patients

**Figure 3:**
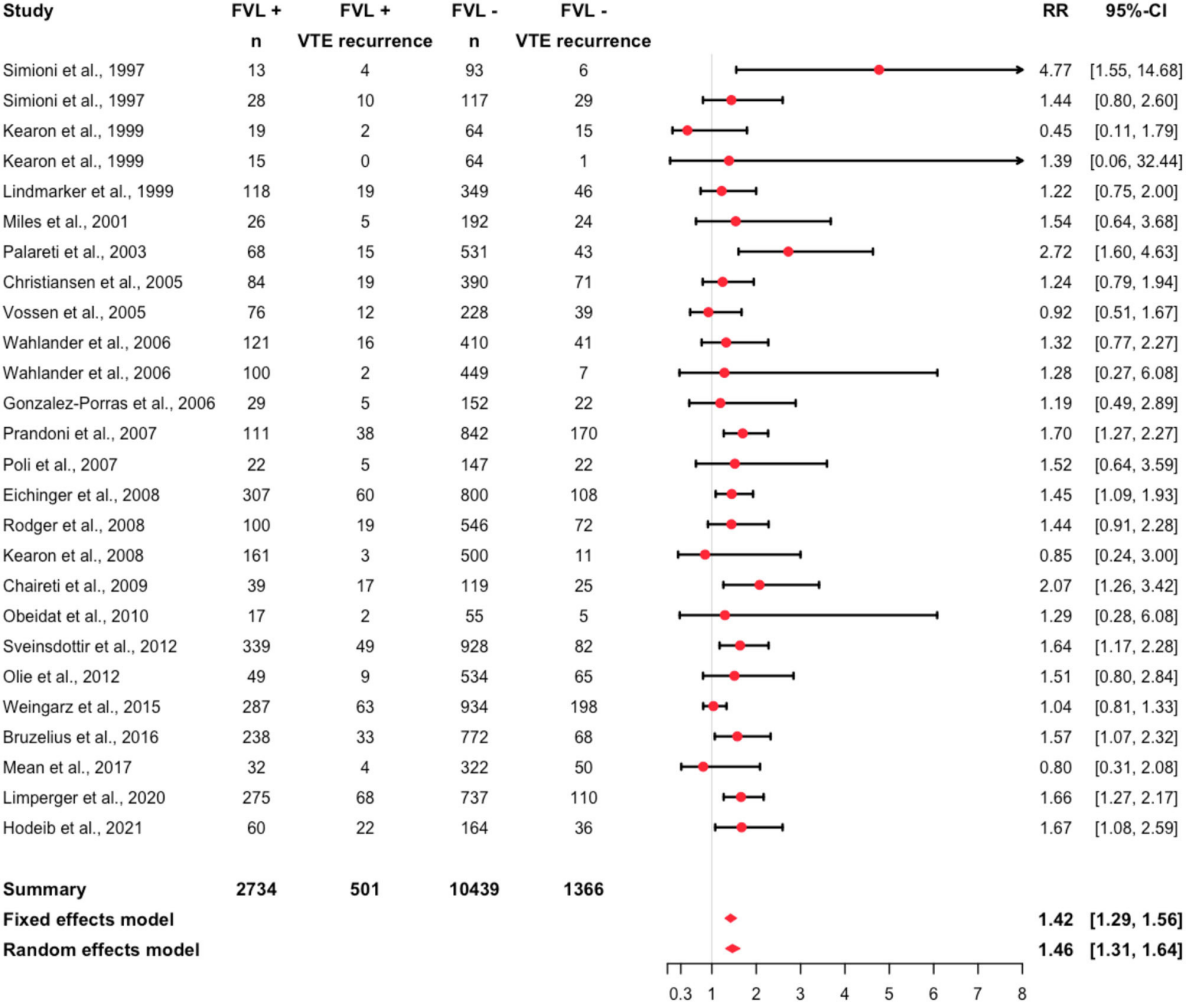
Forest plot summarizing the relative risk of recurrent VTE among heterozygous FVL mutation patients (*I*^2^ = 0.17).

Assessing all studies for potential influential outliers using statistical criteria (105), we identified the study from Franco Moreno et al. (103) (Supplementary Table S2; Supplementary Figure S2). Thus, this study was excluded for the purpose of the overall analysis. A recurrent event was recorded in 1,867 individuals (14%). Recurrent events were observed in 18% of the FVL mutation patients and in 13% of the non-FVL mutation patients. Details are reported in Table 2. The relative risk was 1.46 (95% CI: 1.31, 1.64, *I*^2^ = 0.17; 95% prediction interval 1.10, 1.94) (Figure 3).

In several sensitivity analyses, we assessed the risk in specific subgroups. Among the primary studies, the RR varied between 0.45 (95% CI: 0.11, 1.79) (69) and 4.77 (95% CI: 1.55, 14.68) (67). A RR smaller than one was calculated in four primary studies (48, 69, 70, 73). Focusing on different anticoagulants, the RR was 1.65 (95% CI: 1.33, 2.04) in patients treated with VKA, and 1.28 (95% CI: 0.27, 6.08) in patients treated with DOAC (Supplementary Figure S3). Pooling studies with unprovoked VTE only, the RR was 1.53 (95% CI: 0.99, 2.35) (Supplementary Figure S4). It was 1.47 (95% CI: 1.27, 1.71) in studies including both, patients with provoked and unprovoked VTE. In one study group (67), patients with a first provoked VTE only were analyzed, resulting in a RR of 4.77 (95% CI: 1.55, 14.68). Considering different localizations of the initial event, the RR was 1.29 (95% CI: 0.28, 6.08) in patients with PE (Supplementary Figure S5), and 1.52 (95% CI: 1.2, 1.93) in patients with proximal DVT or PE. It was 1.6 (95% CI: 1.32, 1.95) in patients with proximal DVT/PE or distal DVT. Excluding patients with cancer, the RR was 1.59 (95% CI: 1.27, 1.99) (Supplementary Figure S6). The RR was 1.69 (95% CI: 1.14, 2.51) in studies published after 2011, 1.52 (95% CI: 1.33, 1.75) in studies published between 2001 and 2011, and 1.44 (95% CI: 0.77, 2.68) in studies published before 2001 (Supplementary Figure S7).

### FVL Mutation Testing

Analysis of Helsana health care claims data in Switzerland showed that 46,522 APCR tests and 49,625 polymerase-chain reaction (PCR) for FVL mutation were recorded between 2014 and 2020 (Supplementary Table S3; Supplementary Figure S8). For APCR, the frequency of testing varied between 6,206 (0.1% of the population, 2014) and 7,206 (0.1%, 2016). For PCR, the frequency ranged between 6,793 (0.1%, 2017) and 7,614 (0.1%, 2019). Considering patients having any test, the total number of patients with APCR and/or PCR varied between 9,661 (0.2%, 2017) and 10,614 (0.2%, 2016). The frequency of testing was stable between 2014 and 2020.

## Discussion

We conducted a comprehensive systematic review retrieving all high-quality epidemiological data investigating the association of heterozygous FVL mutation and recurrent VTE. Thirty-one prospective cohort studies were identified and 24 publications summarizing 13,571 patients were included in the meta-analysis. Overall, a 42% increased risk of recurrence was found in patients with heterozygous FVL mutation. Various subgroup analyses did not identify a population with a significantly modified risk. However, a significant proportion of the analyzed Swiss population was tested for FVL mutation each year.

The present work is the most comprehensive systematic review to date. Considering all currently available data, we were able to analyze various subgroups of patients. However, our results are essentially consistent with previous investigations (24–26). Segal et al. (26) included 13 prospective studies summarizing 4,730 patients, reporting an overall odds ratio of 1.56. Marchiori et al. (25) included 10 prospective studies with 3,203 patients concluding on a relative risk of 1.39. Ho et al. (24) summarized two retrospective studies and eight prospective studies, reporting an odds ratio of 1.41. We analyzed a number of patient subgroups (type of anticoagulation, triggering risk factors, VTE localization, presence of cancer, and year of publication) and none of these analyses revealed statistically significant differences in the recurrence risk (Supplementary Figures S3–S7). However, a remarkable higher recurrence risk was reported in the only study including patients with provoked VTE (67). However, this was a small study published in 1997 and the results were never confirmed in other settings.

Our investigation has several strengths. First, we conducted a comprehensive literature search and applied strict inclusion criteria to include high-quality data only. Secondly, we pooled three times more patients compared to the latest systematic review. Thirdly, most of the studies had a low risk of bias and the between-study heterogeneity is low. Fourthly, we were able to conduct several subgroup analyses, thus strengthening the interpretation. Of course, our study has limitations as well. First, inherent with any meta-analytic approach, our investigation relies on data retrieved from primary studies. However, only four studies were estimated to have a high risk of bias. One of those studies was classified as a potentially influential outlier and thus excluded for overall analysis. The remaining three studies affected only 4% of the patients. Thus, we do not believe that this might have influenced our results. Secondly, the number of patients were limited in certain subgroups; patients with provoked VTE, cancer, DOAC, and PE were underrepresented. Thirdly, it was impossible to retrieve separate data for hetero- and homozygous patients in few studies. However, we do not believe that this might have influenced our results because only few patients are included in the large number of patients. Fourthly, one might argue that the proportion of patients with unprovoked VTE varies considerably among studies. However, as long as the between-study heterogeneity was low, this might be regarded as a strength of our study, increasing external validity.

Our data confirm that the presence of FVL mutation represents a minor risk factor only. Compared to the much stronger risk factors unprovoked VTE, proximal DVT/PE, male sex, elevated D-Dimers, high factor VIII plays the presence of FVL mutation only a marginal role (9, 11, 78, 106–109). Consistently, several prediction models for recurrent VTE were developed and FVL mutation was not identified as a relevant predictor in any of the models including clinical characteristics (11, 84, 107, 110, 111). Thus, an important task is to translate this evidence into clinical practice. Determination of FVL mutation shall be challenged and the reimbursement of these analyses might be questioned. However, some authors argue that the presence of FVL mutation might contribute to a significantly elevated risk if combined with other (high risk) thrombophilia. To date, the data supporting this hypothesis are not sufficient. Individual patient-data meta-analyses are a promising tool to study this research question.

## Conclusions

Summarizing all currently available high-quality epidemiological data, the risk of recurrent VTE was only moderately increased. This observation was consistent among various subgroups. Our data confirm that the presence of FVL mutation plays only a marginal role in the risk assessment for recurrent VTE. Efforts should be made to reduce the still very frequent determination in clinical practice.

## Data Availability Statement

The raw data supporting the conclusions of this article will be made available by the authors, without undue reservation.

## Author Contributions

DE developed the search strategy, conducted the literature search, retrieved the data, interpreted the results, wrote the manuscript, and contributed to the study protocol. HN wrote the analysis plan, conducted the analysis, and interpreted the data. BA developed the search strategy and contributed to the literature search. CH collected the data (health care claims) and contributed to the study protocol and the interpretation of the data. MN developed the study protocol, conducted the literature search, contributed to the analysis plan, interpreted the results, and wrote the manuscript. All authors contributed to and approved the final manuscript.

## Funding

MN was supported by a research grant of the Swiss National Science Foundation (#179334).

## Conflict of Interest

The authors declare that the research was conducted in the absence of any commercial or financial relationships that could be construed as a potential conflict of interest.

## Publisher's Note

All claims expressed in this article are solely those of the authors and do not necessarily represent those of their affiliated organizations, or those of the publisher, the editors and the reviewers. Any product that may be evaluated in this article, or claim that may be made by its manufacturer, is not guaranteed or endorsed by the publisher.
