# Supplementary material for: Current Knowledge on Factor V Leiden Mutation as a Risk Factor for Recurrent Venous Thromboembolism: A Systematic Review and Meta-Analysis
Source: Front Cardiovasc Med. 2022 Apr 7;9:883986. doi: 10.3389/fcvm.2022.883986 (PMC9021545; doi:10.3389/fcvm.2022.883986)
Supplement: Supplementary file 1 [file Data_Sheet_1.PDF]

Supplementary Material

Table of contents

1 Supplementary Data 2

1.1 List 1: Search strategy for MEDLINE and EMBASE 2

1.2 List 2: Questions and levels of the adapted NOS scale. 4

2 Supplementary Figures and Tables 6

2.1 Figure S1: Funnel plot. Egger' test does not indicate the presence of a relevant asymmetry. 6

2.2 Table S1: Methodological quality of individual studies. 7

2.3 Table S2: Leave one out analysis 9

2.4 Figure S2: Influence analysis 10

2.5 Figure S3: Subgroup analysis; type of anticoagulation 11

2.6 Figure S4: Subgroup analysis; trigger of initial event 12

2.7 Figure S5: Subgroup analysis; localization of initial event 13

2.8 Figure S6: Subgroup analysis; with and without cancer patients 14

2.9 Figure S7: Subgroup analysis; year of publication 15

2.10 Table S3: Frequency of testing for FVL mutation in the Swiss health care system 16

2.11 Figure S8: Variation in FVL mutation testing from 2014 to 2020 17

3 References 18

1 Supplementary Data

1.1 List 1: Search strategy for MEDLINE and EMBASE

1 Activated Protein C Resistance/

2 exp Factor V/ or blood clotting factor 5/ or blood clotting factor 5 leiden/

3 Thrombophilia/co, cn, ge, pp, bl

4 Polymorphism, Single Nucleotide/ or single nucleotide polymorphism/

5 factor v leiden mutation.ab,kw,ti.

6 "factor v leiden".ab,kw,ti.

7 FVL.ab,kw,ti.

8 Activated protein C Resistance.ab,kw,ti.

9 APCR.ab,kw,ti.

10 R506Q.ab,kw,ti.

11 G1691A.ab,kw,ti.

12 single nucleotide polymorphism?.ab,kw,ti.

13 SNP.ab,kw,ti.

14 1 or 2 or 3 or 4 or 5 or 6 or 7 or 8 or 9 or 10 or 11 or 12 or 13

15 thromboembolism/ or venous thromboembolism/ or thrombosis/ or venous thrombosis/ or vein thrombosis/ or deep vein thrombosis/

16 Pulmonary Embolism/ or lung embolism/

17 "venous thrombo*".ab,kw,ti.

18 "Deep vein thrombo*".ab,kw,ti.

19 pulmonary embolism.ab,kw,ti.

20 "pulmonary embolis*".ab,kw,ti.

21 VTE.ab,kw,ti.

22 DVT.ab,kw,ti.

23 PE.ab,kw,ti.

24 "lung embolis*".ab,kw,ti.

25 15 or 16 or 17 or 18 or 19 or 20 or 21 or 22 or 23 or 24

26 cohort studies/ or follow-up studies/ or longitudinal studies/ or prospective studies/ or longitudinal study/ or cohort analysis/ or follow up/ or prospective study/

27 cohort stud*.tw.

28 cohort analysis.ab,kw,ti.

29 "major clinical stud*".ab,kw,ti.

30 controlled study.ab,kw,ti.

31 26 or 27 or 28 or 29 or 30

32 14 and 25 and 31

33 remove duplicates from 32

1.2 List 2: Questions and levels of the adapted NOS scale.

Selection

D1) Representativeness of patients with FVL in the community

a) truly representative: Recruitment without knowledge of FVL. (+)

b) Selection according to the presence of FVL (-)

c) no description of the derivation of the cohort (?)

D2) Selection of the non-exposed cohort

a) drawn from the same study population as the exposed cohort (+)

b) drawn from a different source (-)

c) no description of the derivation of the non-exposed cohort (?)

D3) Ascertainment of exposure: Testing of FVL

a) APCR or/and PCR tested at baseline (+)

b) Patient documentation (+)

c) no description (?)

D4) Demonstration that recurrent VTE was not present at start of study

a) Objectively confirmed, described how recurrent VTE was handled to differentiate from initial VTE (+)

b) Objectively confirmed, unclear how recurrent VTE were handled to differentiate from initial VTE (?)

c) Not described, Questionnaire (-)

Comparability

D5) Comparability of cohorts on the basis of the design or analysis

a) Analyses were adjusted for confounders (+)

b) Analyses were not adjusted for confounders (-)

c) Not described (?)

Outcome

D6) Assessment of outcome: objectively confirmed VTE

a) independent blind assessment (+)

b) record linkage (+)

c) self-report (-)

d) no description (?)

D7) Acceptable length of follow-up: 2 years

a) yes (+)

b) no (-)

c) Unclear (?)

D8) Complete follow up

a) complete follow up (+)

b) Small number lost < 10 % (+)

c) follow up rate < 90% (-)

d) no statement (?)

2 Supplementary Figures and Tables

2.1 Figure S1: Funnel plot. Egger' test does not indicate the presence of a relevant asymmetry.

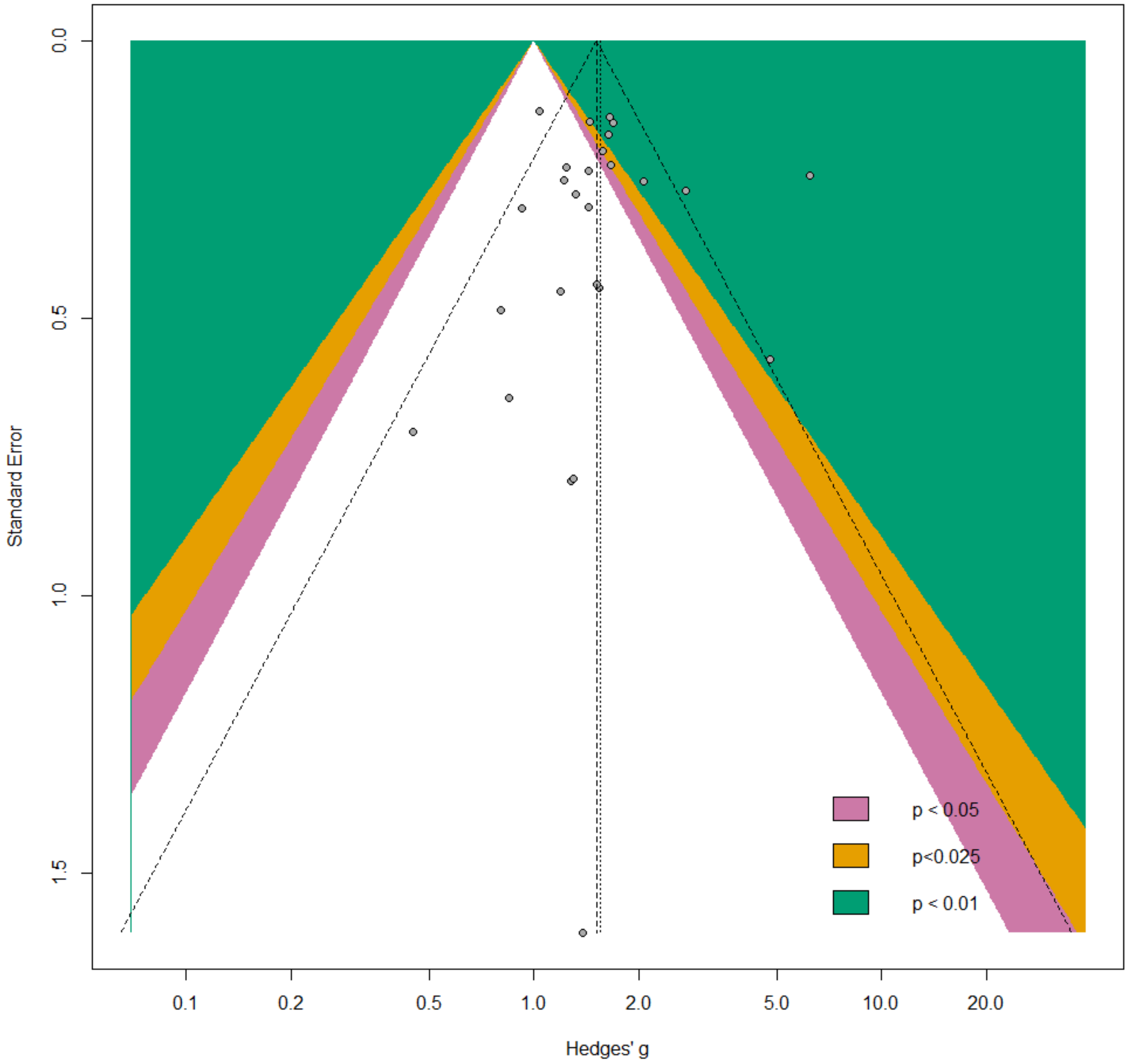

2.2 Table S1: Methodological quality of individual studies.

Risk of bias domains

Study	D1	D2	D3	D4	D5	D6	D7	D8	Overall
1	+	+	+	-	+	+	+	-	+
2	+	+	+	+	+	+	X	+	+
3	+	+	X	+	+	+	+	+	+
4	+	+	+	-	+	+	+	+	+
5	+	+	+	+	+	+	X	+	+
6	+	+	+	-	+	+	+	+	+
7	X	X	+	-	+	+	+	+	X
8	+	+	+	+	+	+	X	-	+
9	+	+	+	-	+	-	+	-	X
10	+	+	+	+	+	+	+	+	+
11	+	+	+	+	+	+	+	+	+
12	+	+	+	+	+	+	+	+	+
13	+	+	+	+	+	+	X	+	+
14	+	+	+	+	X	+	+	+	+
15	+	+	+	-	+	+	+	+	+
16	+	+	+	-	X	-	X	X	X
17	+	+	+	-	+	+	+	+	+
18	+	+	+	+	+	X	+	+	+
19	+	+	+	+	+	+	+	+	+
20	+	+	+	-	+	+	+	+	+
21	+	+	-	-	+	+	X	-	X
22	+	+	+	-	+	+	+	-	+
23	+	+	+	-	+	+	+	-	+
24	+	+	+	-	+	+	+	-	+

D1: Selection of FVL patients
D2: Selection of non FVL patients
D3: Ascertainment of FVL
D4: Differentiation between initial and recurrent VTE
D5: Comparability
D6: Assessment of recurrent VTE
D7: Length of follow up more than 2 years
D8: Complete follow up

Judgement
+ Low
- Unclear
X High
! Critical

1. Simioni 1997(1)
2. Kearon 1999(2)

3. Lindmarker 1999(3)
4. Miles 2001(4)
5. Palareti 2003(5)
6. Christinasen 2005(6)
7. Vossen 2005(7)
8. Wahlander 2006(8)
9. Gonzalez-Porras 2006(9)
10. Prandoni 2007(10)
11. Poli 2007(11)
12. Eichinger 2008(12)
13. Rodger 2008(13)
14. Kearon 2008(14)
15. Chaireti 2009(15)
16. Obeidat 2010(16)
17. Sveinsdottir 2012(17)
18. Olie 2012(18)
19. Weingarz 2015(19)
20. Moreno 2016(20)
21. Bruzelius 2016(21)
22. Mean 2017(22)
23. Limperger 2020(23)
24. Hodeib 2021(24)

2.3 Table S2: Leave one out analysis

Study omitted	DFFITS	Cook's distance	Hat	Influence
1 - Simioni, 1997	0.220	0.048	0.018	
2 - Simioni, 1997	-0.170	0.001	0.039	
3 - Kearon, 1999	-0.183	0.033	0.013	
4 - Kearon, 1999	-0.003	0.000	0.003	
5 - Lindmarker, 1999	-0.123	0.016	0.045	
6 - Miles, 2001	0.003	0.000	0.025	
7 - Palareti, 2003	0.284	0.078	0.043	
8 - Christiansen, 2005	-0.121	0.015	0.048	
9 - Vossen, 2005	-0.247	0.060	0.039	
10 - Wahlander, 2006	-0.072	0.005	0.042	
11 - Wahlander, 2006	-0.021	0.000	0.010	
12 - Gonzales-Porras, 2006	-0.073	0.005	0.025	
13 - Prandoni, 2007	0.072	0.006	0.060	
14 - Poli, 2007	-0.001	0.000	0.026	
15 - Eichinger, 2008	-0.037	0.001	0.060	
16 - Rodger, 2008	-0.032	0.001	0.048	
17 - Kearon, 2008	-0.103	0.011	0.015	
18 - Chaireti, 2009	0.159	0.011	0.015	
19 - Obeidat, 2010	-0.020	0.000	0.010	
20 - Sveinsdottir, 2012	0.046	0.002	0.057	
21 - Olie, 2012	-0.005	0.000	0.036	
22 - Weingarz, 2015	-0.338	0.107	0.063	
23 - Moreno, 2016	1.028	0.481	0.046	*
24 - Bruzelius, 2016	0.019	0.000	0.053	
25 - Mean, 2017	-0.174	0.030	0.022	
26 - Limperger, 2020	0.057	0.004	0.061	
27 - Hodeib, 2021	0.052	0.003	0.049	

Cut-offs:

- The studentized residuals are outside -1 and 1
- The DFFITS value is larger than 3(pk-p)
- The area of the lower tail of a Chi-square distribution with p degrees of freedom cut off by the Cook's distance is larger than 50%

P = number of model coefficients; k = number of studies

2.4 Figure S2: Influence analysis

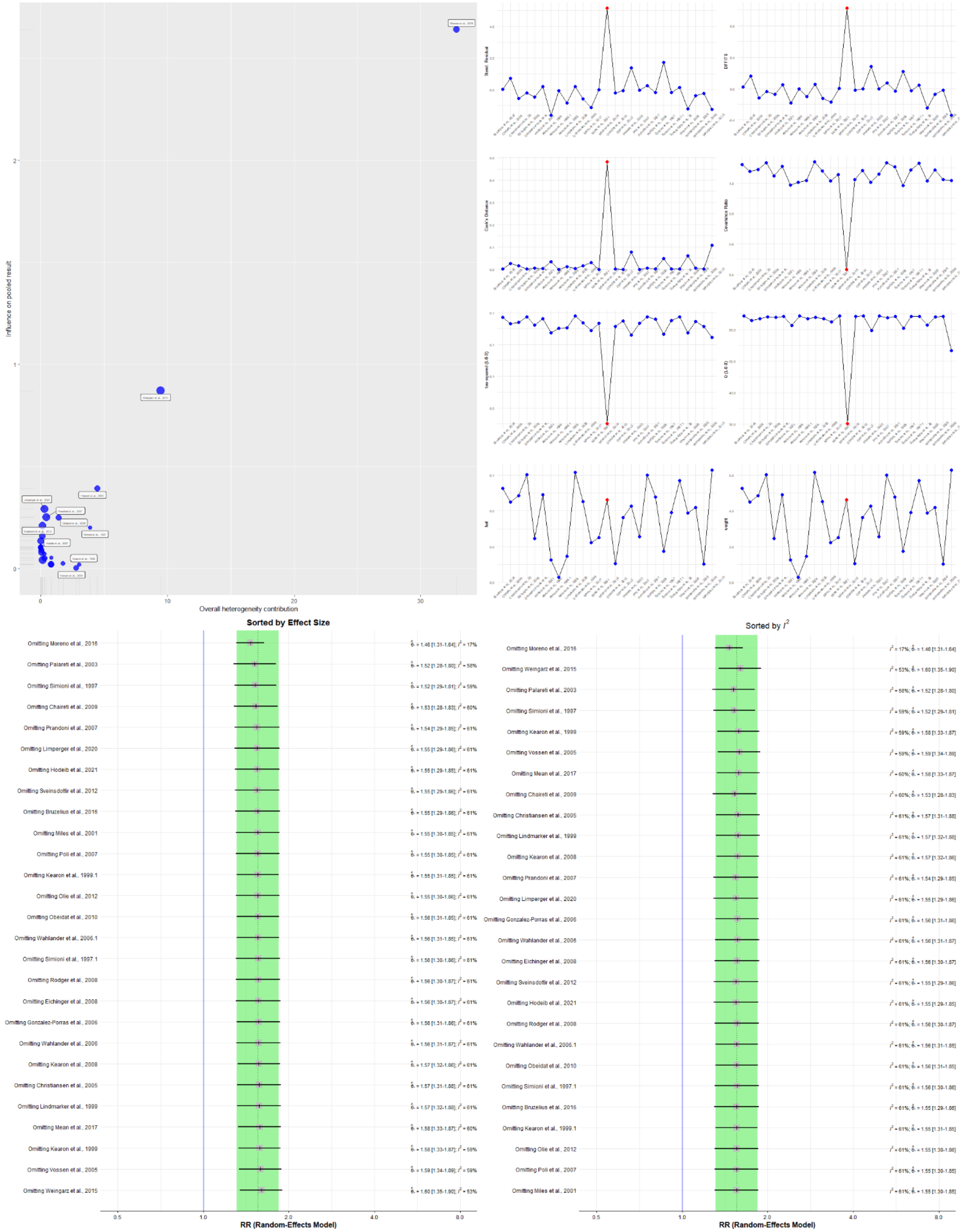

2.5 Figure S3: Subgroup analysis; type of anticoagulation

Study	FVL + n	FVL + VTE recurrence	FVL - n	FVL - VTE recurrence	RR	95%-CI	No. of patients
**Mixed / Not mentioned**							
Miles et al., 2001	26	5	192	24	1.54	[0.64, 3.68]	218
Vossen et al., 2005	76	12	228	39	0.92	[0.51, 1.67]	304
Obeidat et al., 2010	17	2	55	5	1.29	[0.28, 6.08]	72
Weingarz et al., 2015	287	63	934	198	1.04	[0.81, 1.33]	1221
Bruzelius et al., 2016	238	33	772	68	1.57	[1.07, 2.32]	1010
Limperger et al., 2020	275	68	737	110	1.66	[1.27, 2.17]	1012
**Random effects model**	**919**	**183**	**2918**	**444**	**1.31**	**[1.02, 1.69]**	**3837**
**DOAC**							
Wahlander et al., 2006	100	2	449	7	1.28	[0.27, 6.08]	549
**Random effects model**	**100**	**2**	**449**	**7**	**1.28**	**[0.27, 6.08]**	**549**
**VKA**							
Simioni et al., 1997	13	4	93	6	4.77	[1.55, 14.68]	106
Simioni et al., 1997	28	10	117	29	1.44	[0.80, 2.60]	145
Kearon et al., 1999	19	2	64	15	0.45	[0.11, 1.79]	83
Kearon et al., 1999	15	0	64	1	1.39	[0.06, 32.44]	79
Lindmarker et al., 1999	118	19	349	46	1.22	[0.75, 2.00]	467
Palareti et al., 2003	68	15	531	43	2.72	[1.60, 4.63]	599
Christiansen et al., 2005	84	19	390	71	1.24	[0.79, 1.94]	474
Wahlander et al., 2006	121	16	410	41	1.32	[0.77, 2.27]	531
Gonzalez-Porras et al., 2006	29	5	152	22	1.19	[0.49, 2.89]	181
Prandoni et al., 2007	111	38	842	170	1.70	[1.27, 2.27]	953
Poli et al., 2007	22	5	147	22	1.52	[0.64, 3.59]	169
Eichinger et al., 2008	307	60	800	108	1.45	[1.09, 1.93]	1107
Rodger et al., 2008	100	19	546	72	1.44	[0.91, 2.28]	646
Kearon et al., 2008	161	3	500	11	0.85	[0.24, 3.00]	661
Chaireti et al., 2009	39	17	119	25	2.07	[1.26, 3.42]	158
Sveinsdottir et al., 2012	339	49	928	82	1.64	[1.17, 2.28]	1267
Olie et al., 2012	49	9	534	65	1.51	[0.80, 2.84]	583
Moreno et al., 2016	106	45	292	20	6.20	[3.84, 9.99]	398
Mean et al., 2017	32	4	322	50	0.80	[0.31, 2.08]	354
Hodeib et al., 2021	60	22	164	36	1.67	[1.08, 2.59]	224
**Random effects model**	**1821**	**361**	**7364**	**935**	**1.65**	**[1.33, 2.04]**	**9185**

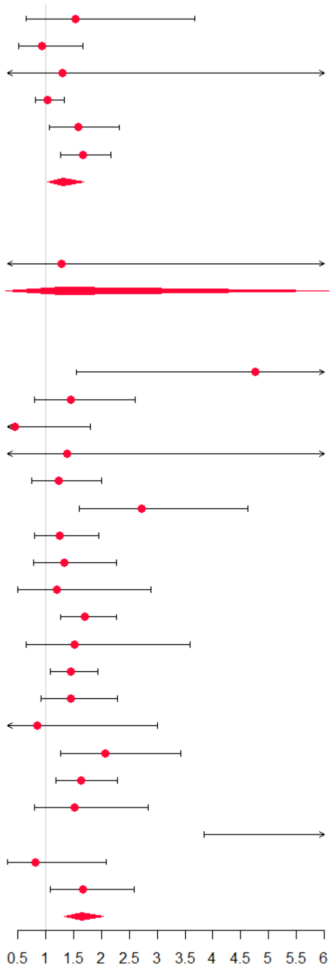

2.6 Figure S4: Subgroup analysis; trigger of initial event

Study	FVL + n	FVL + VTE recurrence	FVL - n	FVL - VTE recurrence	RR	95%-CI	No. of patients
**Provoked**							
Simioni et al., 1997	13	4	93	6	4.77	[1.55, 14.68]	106
**Random effects model**	**13**	**4**	**93**	**6**	**4.77**	**[1.55, 14.68]**	**106**
**Mixed**							
Lindmarker et al., 1999	118	19	349	46	1.22	[0.75, 2.00]	467
Miles et al., 2001	26	5	192	24	1.54	[0.64, 3.68]	218
Palareti et al., 2003	68	15	531	43	2.72	[1.60, 4.63]	599
Christiansen et al., 2005	84	19	390	71	1.24	[0.79, 1.94]	474
Vossen et al., 2005	76	12	228	39	0.92	[0.51, 1.67]	304
Wahlander et al., 2006	121	16	410	41	1.32	[0.77, 2.27]	531
Wahlander et al., 2006	100	2	449	7	1.28	[0.27, 6.08]	549
Gonzalez-Porras et al., 2006	29	5	152	22	1.19	[0.49, 2.89]	181
Prandoni et al., 2007	111	38	842	170	1.70	[1.27, 2.27]	953
Poli et al., 2007	22	5	147	22	1.52	[0.64, 3.59]	169
Chaireti et al., 2009	39	17	119	25	2.07	[1.26, 3.42]	158
Obeidat et al., 2010	17	2	55	5	1.29	[0.28, 6.08]	72
Sveinsdottir et al., 2012	339	49	928	82	1.64	[1.17, 2.28]	1267
Weingarz et al., 2015	287	63	934	198	1.04	[0.81, 1.33]	1221
Bruzelius et al., 2016	238	33	772	68	1.57	[1.07, 2.32]	1010
Limperger et al., 2020	275	68	737	110	1.66	[1.27, 2.17]	1012
**Random effects model**	**1950**	**368**	**7235**	**973**	**1.47**	**[1.27, 1.71]**	**9185**
**Unprovoked**							
Simioni et al., 1997	28	10	117	29	1.44	[0.80, 2.60]	145
Kearon et al., 1999	19	2	64	15	0.45	[0.11, 1.79]	83
Kearon et al., 1999	15	0	64	1	1.39	[0.06, 32.44]	79
Eichinger et al., 2008	307	60	800	108	1.45	[1.09, 1.93]	1107
Rodger et al., 2008	100	19	546	72	1.44	[0.91, 2.28]	646
Kearon et al., 2008	161	3	500	11	0.85	[0.24, 3.00]	661
Olie et al., 2012	49	9	534	65	1.51	[0.80, 2.84]	583
Moreno et al., 2016	106	45	292	20	6.20	[3.84, 9.99]	398
Mean et al., 2017	32	4	322	50	0.80	[0.31, 2.08]	354
Hodeib et al., 2021	60	22	164	36	1.67	[1.08, 2.59]	224
**Random effects model**	**877**	**174**	**3403**	**407**	**1.53**	**[0.99, 2.35]**	**4280**

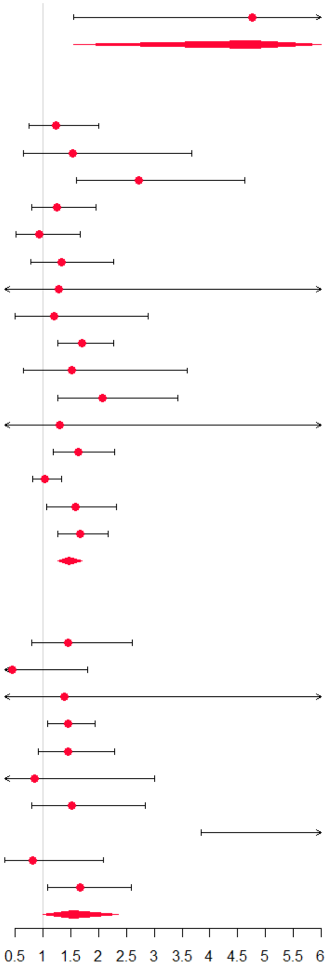

2.7 Figure S5: Subgroup analysis; localization of initial event

Study	FVL + n	FVL + VTE recurrence	FVL - n	FVL - VTE recurrence	RR	95%-CI	No. of patients
**PE**							
Obeidat et al., 2010	17	2	55	5	1.29	[0.28, 6.08]	72
**Random effects model**	**17**	**2**	**55**	**5**	**1.29**	**[0.28, 6.08]**	**72**
**Mixed**							
Simioni et al., 1997	13	4	93	6	4.77	[1.55, 14.68]	106
Simioni et al., 1997	28	10	117	29	1.44	[0.80, 2.60]	145
Lindmarker et al., 1999	118	19	349	46	1.22	[0.75, 2.00]	467
Miles et al., 2001	26	5	192	24	1.54	[0.64, 3.68]	218
Palareti et al., 2003	68	15	531	43	2.72	[1.60, 4.63]	599
Christiansen et al., 2005	84	19	390	71	1.24	[0.79, 1.94]	474
Vossen et al., 2005	76	12	228	39	0.92	[0.51, 1.67]	304
Wahlander et al., 2006	121	16	410	41	1.32	[0.77, 2.27]	531
Wahlander et al., 2006	100	2	449	7	1.28	[0.27, 6.08]	549
Gonzalez-Porras et al., 2006	29	5	152	22	1.19	[0.49, 2.89]	181
Poli et al., 2007	22	5	147	22	1.52	[0.64, 3.59]	169
Eichinger et al., 2008	307	60	800	108	1.45	[1.09, 1.93]	1107
Chaireti et al., 2009	39	17	119	25	2.07	[1.26, 3.42]	158
Sveinsdottir et al., 2012	339	49	928	82	1.64	[1.17, 2.28]	1267
Olie et al., 2012	49	9	534	65	1.51	[0.80, 2.84]	583
Weingarz et al., 2015	287	63	934	198	1.04	[0.81, 1.33]	1221
Moreno et al., 2016	106	45	292	20	6.20	[3.84, 9.99]	398
Bruzelius et al., 2016	238	33	772	68	1.57	[1.07, 2.32]	1010
Mean et al., 2017	32	4	322	50	0.80	[0.31, 2.08]	354
Limperger et al., 2020	275	68	737	110	1.66	[1.27, 2.17]	1012
Hodeib et al., 2021	60	22	164	36	1.67	[1.08, 2.59]	224
**Random effects model**	**2417**	**482**	**8660**	**1112**	**1.60**	**[1.32, 1.95]**	**11077**
**Proximal DVT/ PE**							
Kearon et al., 1999	19	2	64	15	0.45	[0.11, 1.79]	83
Kearon et al., 1999	15	0	64	1	1.39	[0.06, 32.44]	79
Prandoni et al., 2007	111	38	842	170	1.70	[1.27, 2.27]	953
Rodger et al., 2008	100	19	546	72	1.44	[0.91, 2.28]	646
Kearon et al., 2008	161	3	500	11	0.85	[0.24, 3.00]	661
**Random effects model**	**406**	**62**	**2016**	**269**	**1.52**	**[1.20, 1.93]**	**2422**

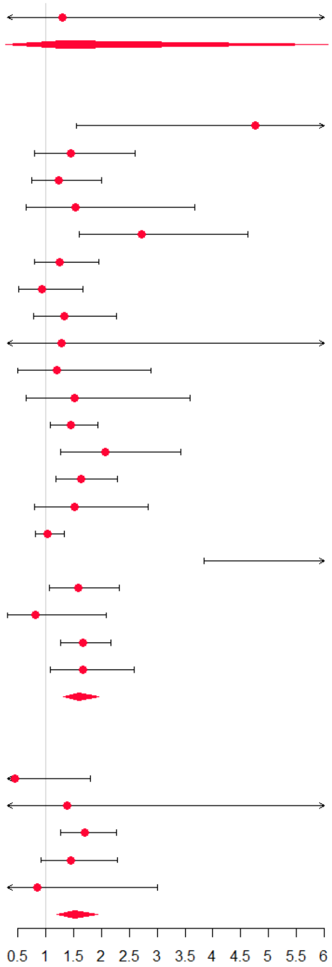

2.8 Figure S6: Subgroup analysis; with and without cancer patients

Study	FVL + n	FVL + VTE recurrence	FVL - n	FVL - VTE recurrence	RR	95%-CI	No. of patients
**No cancer patients**							
Simioni et al., 1997	13	4	93	6	4.77	[1.55, 14.68]	106
Simioni et al., 1997	28	10	117	29	1.44	[0.80, 2.60]	145
Kearon et al., 1999	19	2	64	15	0.45	[0.11, 1.79]	83
Kearon et al., 1999	15	0	64	1	1.39	[0.06, 32.44]	79
Lindmarker et al., 1999	118	19	349	46	1.22	[0.75, 2.00]	467
Christiansen et al., 2005	84	19	390	71	1.24	[0.79, 1.94]	474
Gonzalez-Porras et al., 2006	29	5	152	22	1.19	[0.49, 2.89]	181
Prandoni et al., 2007	111	38	842	170	1.70	[1.27, 2.27]	953
Poli et al., 2007	22	5	147	22	1.52	[0.64, 3.59]	169
Eichinger et al., 2008	307	60	800	108	1.45	[1.09, 1.93]	1107
Rodger et al., 2008	100	19	546	72	1.44	[0.91, 2.28]	646
Kearon et al., 2008	161	3	500	11	0.85	[0.24, 3.00]	661
Olie et al., 2012	49	9	534	65	1.51	[0.80, 2.84]	583
Moreno et al., 2016	106	45	292	20	6.20	[3.84, 9.99]	398
Bruzelius et al., 2016	238	33	772	68	1.57	[1.07, 2.32]	1010
Mean et al., 2017	32	4	322	50	0.80	[0.31, 2.08]	354
Limperger et al., 2020	275	68	737	110	1.66	[1.27, 2.17]	1012
Hodeib et al., 2021	60	22	164	36	1.67	[1.08, 2.59]	224
**Random effects model**	**1767**	**365**	**6885**	**922**	**1.59**	**[1.27, 1.99]**	**8652**
**Mixed**							
Palareti et al., 2003	68	15	531	43	2.72	[1.60, 4.63]	599
Vossen et al., 2005	76	12	228	39	0.92	[0.51, 1.67]	304
Wahlander et al., 2006	121	16	410	41	1.32	[0.77, 2.27]	531
Wahlander et al., 2006	100	2	449	7	1.28	[0.27, 6.08]	549
Chaireti et al., 2009	39	17	119	25	2.07	[1.26, 3.42]	158
Sveinsdottir et al., 2012	339	49	928	82	1.64	[1.17, 2.28]	1267
Weingarz et al., 2015	287	63	934	198	1.04	[0.81, 1.33]	1221
**Random effects model**	**1030**	**174**	**3599**	**435**	**1.48**	**[1.09, 2.01]**	**4629**
**Not mentioned**							
Miles et al., 2001	26	5	192	24	1.54	[0.64, 3.68]	218
Obeidat et al., 2010	17	2	55	5	1.29	[0.28, 6.08]	72
**Random effects model**	**43**	**7**	**247**	**29**	**1.48**	**[0.69, 3.15]**	**290**

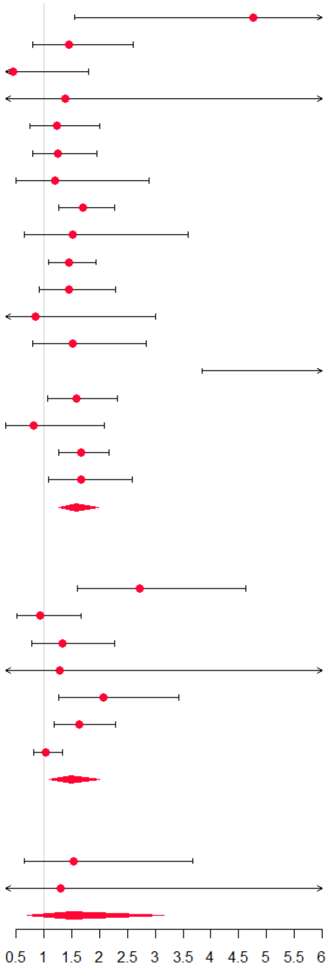

2.9 Figure S7: Subgroup analysis; year of publication

Study	FVL + n	FVL + TE recurrence	FVL - n	FVL - TE recurrence	RR	95%-CI	No. of patients
**< 2000**							
Simioni et al., 1997	13	4	93	6	4.77	[1.55, 14.68]	106
Simioni et al., 1997	28	10	117	29	1.44	[0.80, 2.60]	145
Kearon et al., 1999	19	2	64	15	0.45	[0.11, 1.79]	83
Kearon et al., 1999	15	0	64	1	1.39	[0.06, 32.44]	79
Lindmarker et al., 1999	118	19	349	46	1.22	[0.75, 2.00]	467
**Random effects model**	**193**	**35**	**687**	**97**	**1.44**	**[0.77, 2.68]**	**880**
**2000 - 2010**							
Miles et al., 2001	26	5	192	24	1.54	[0.64, 3.68]	218
Palareti et al., 2003	68	15	531	43	2.72	[1.60, 4.63]	599
Christiansen et al., 2005	84	19	390	71	1.24	[0.79, 1.94]	474
Vossen et al., 2005	76	12	228	39	0.92	[0.51, 1.67]	304
Wahlander et al., 2006	121	16	410	41	1.32	[0.77, 2.27]	531
Wahlander et al., 2006	100	2	449	7	1.28	[0.27, 6.08]	549
Gonzalez-Porras et al., 2006	29	5	152	22	1.19	[0.49, 2.89]	181
Prandoni et al., 2007	111	38	842	170	1.70	[1.27, 2.27]	953
Poli et al., 2007	22	5	147	22	1.52	[0.64, 3.59]	169
Eichinger et al., 2008	307	60	800	108	1.45	[1.09, 1.93]	1107
Rodger et al., 2008	100	19	546	72	1.44	[0.91, 2.28]	646
Kearon et al., 2008	161	3	500	11	0.85	[0.24, 3.00]	661
Chaireti et al., 2009	39	17	119	25	2.07	[1.26, 3.42]	158
Obeidat et al., 2010	17	2	55	5	1.29	[0.28, 6.08]	72
**Random effects model**	**1261**	**218**	**5361**	**660**	**1.52**	**[1.33, 1.75]**	**6622**
**> 2010**							
Sveinsdottir et al., 2012	339	49	928	82	1.64	[1.17, 2.28]	1267
Olie et al., 2012	49	9	534	65	1.51	[0.80, 2.84]	583
Weingarz et al., 2015	287	63	934	198	1.04	[0.81, 1.33]	1221
Moreno et al., 2016	106	45	292	20	6.20	[3.84, 9.99]	398
Bruzelius et al., 2016	238	33	772	68	1.57	[1.07, 2.32]	1010
Mean et al., 2017	32	4	322	50	0.80	[0.31, 2.08]	354
Limperger et al., 2020	275	68	737	110	1.66	[1.27, 2.17]	1012
Hodeib et al., 2021	60	22	164	36	1.67	[1.08, 2.59]	224
**Random effects model**	**1386**	**293**	**4683**	**629**	**1.69**	**[1.14, 2.51]**	**6069**

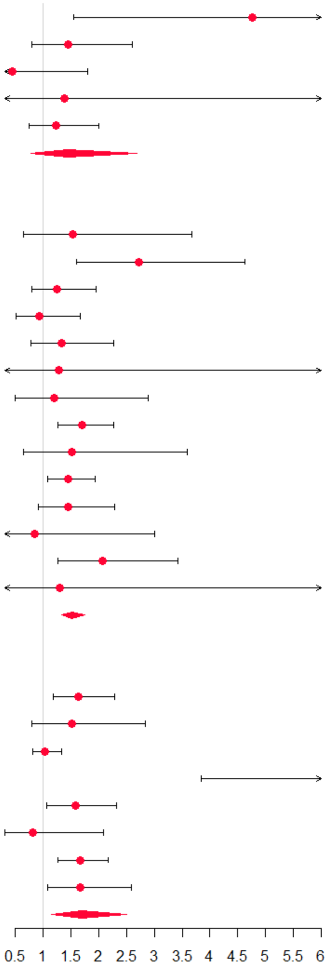

2.10 Table S3: Frequency of testing for FVL mutation in the Swiss health care system

		2014	2015	2016	2017	2018	2019	2020
		n (%)*	n (%)*	n (%)*	n (%)*	n (%)*	n (%)*	n (%)*
APCR (#1086.00)	patients	6028 (0.1)	6368 (0.1)	7049 (0.1)	6285 (0.1)	6156 (0.1)	6685 (0.1)	6601 (0.1)
	total	6206	6574	7206	6484	6298	6944	6810
PCR FVL mutation (#6200.64)	patients	6915 (0.1)	6976 (0.1)	6905 (0.1)	6617 (0.1)	6697 (0.1)	7218 (0.1)	6864 (0.1)
	total	7118	7187	6980	6793	6923	7614	7010
Total number of patients with APCR and/or PCR FVL		9900 (0.2)	10249 (0.2)	10614 (0.2)	9661 (0.2)	9706 (0.1)	9976 (0.2)	9768 (0.1)

* percentage of the Swiss population

2.11 Figure S8: Variation in FVL mutation testing from 2014 to 2020

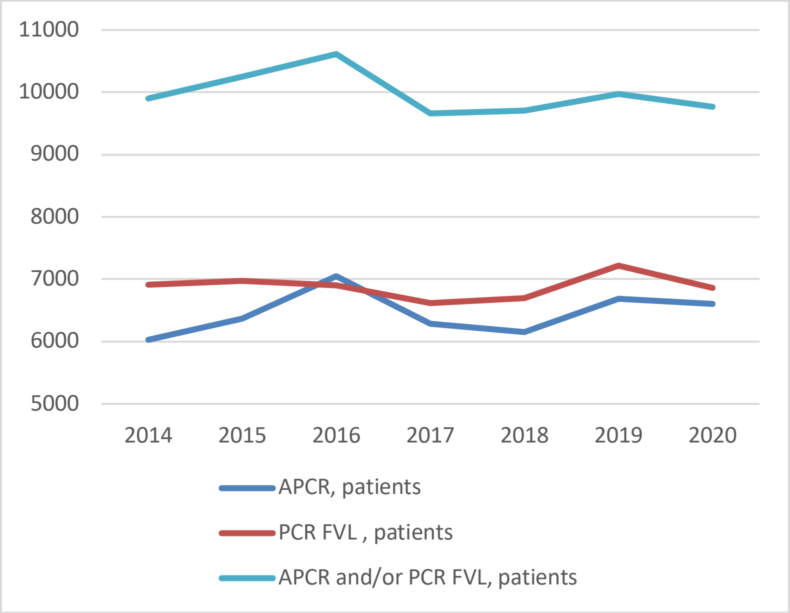

3 References

1. Simioni P, Prandoni P, Lensing AW, Scudeller A, Sardella C, Prins MH, et al. The risk of recurrent venous thromboembolism in patients with an Arg506-->Gln mutation in the gene for factor V (factor V Leiden). N Engl J Med. 1997;336(6):399-403.

2. Kearon C, Gent M, Hirsh J, Weitz J, Kovacs MJ, Anderson DR, et al. A comparison of three months of anticoagulation with extended anticoagulation for a first episode of idiopathic venous thromboembolism. N Engl J Med. 1999;340(12):901-7.

3. Lindmarker P, Schulman S, Sten-Linder M, Wiman B, Egberg N, Johnsson H. The risk of recurrent venous thromboembolism in carriers and non-carriers of the G1691A allele in the coagulation factor V gene and the G20210A allele in the prothrombin gene. Thrombosis and Haemostasis. 1999;81(5):684-9.

4. Miles JS, Miletich JP, Goldhaber SZ, Hennekens CH, Ridker PM. G20210A mutation in the prothrombin gene and the risk of recurrent venous thromboembolism. Journal of the American College of Cardiology. 2001;37(1):215-8.

5. Palareti G, Legnani C, Cosmi B, Valdre L, Lunghi B, Bernardi F, et al. Predictive value of D-dimer test for recurrent venous thromboembolism after anticoagulation withdrawal in subjects with a previous idiopathic event and in carriers of congenital thrombophilia. Circulation. 2003;108(3):313-8.

6. Christiansen SC, Cannegieter SC, Koster T, Vandenbroucke JP, Rosendaal FR. Thrombophilia, clinical factors, and recurrent venous thrombotic events. Jama. 2005;293(19):2352-61.

7. Vossen CY, Walker ID, Svensson P, Souto JC, Scharrer I, Preston FE, et al. Recurrence rate after a first venous thrombosis in patients with familial thrombophilia. Arterioscler Thromb Vasc Biol. 2005;25(9):1992-7.

8. Wahlander K, Eriksson H, Lundstrom T, Clason SB, Wall U, Nystrom P, et al. Risk of recurrent venous thromboembolism or bleeding in relation to thrombophilic risk factors in patients receiving ximelagatran or placebo for long-term secondary prevention of venous thromboembolism. Br J Haematol. 2006;133(1):68-77.

9. Gonzalez-Porras JR, Garcia-Sanz R, Alberca I, Lopez ML, Balanzategui A, Gutierrez O, et al. Risk of recurrent venous thrombosis in patients with G20210A mutation in the prothrombin gene or factor V Leiden mutation. Blood Coagulation and Fibrinolysis. 2006;17(1):23-8.

10. Prandoni P, Noventa F, Ghirarduzzi A, Pengo V, Bernardi E, Pesavento R, et al. The risk of recurrent venous thromboembolism after discontinuing anticoagulation in patients with acute proximal deep vein thrombosis or pulmonary embolism. A prospective cohort study in 1,626 patients. Haematologica. 2007;92(2):199-205+III-IV.

11. Poli D, Antonucci E, Ciuti G, Abbate R, Prisco D. Anticoagulation quality and the risk of recurrence of venous thromboembolism [3]. Thrombosis and Haemostasis. 2007;98(5):1148-50.

12. Eichinger S, Hron G, Bialonczyk C, Hirschl M, Minar E, Wagner O, et al. Overweight, obesity, and the risk of recurrent venous thromboembolism. Arch Intern Med. 2008;168(15):1678-83.

13. Rodger MA, Kahn SR, Wells PS, Anderson DA, Chagnon I, Le Gal G, et al. Identifying unprovoked thromboembolism patients at low risk for recurrence who can discontinue anticoagulant therapy. Cmaj. 2008;179(5):417-26.

14. Kearon C, Julian JA, Kovacs MJ, Anderson DR, Wells P, MacKinnon B, et al. Influence of thrombophilia on risk of recurrent venous thromboembolism while on warfarin: Results from a randomized trial. Blood. 2008;112(12):4432-6.

15. Chaireti R, Jennersjo C, Lindahl TL. Factor V Leiden and thrombin generation and risk of recurrent thrombotic episodes in a patient cohort with venous thromboembolism. The list study. Journal of Thrombosis and Haemostasis. 2009;7(S2):738.

16. Obeidat NM. The effect of genetically related risk factors on the recurrence rate of acute pulmonary embolism in a Tertiary Teaching Hospital in Jordan. Jordan Medical Journal. 2010;44(4):398-403.

17. Sveinsdottir SV, Saemundsson Y, Isma N, Gottsater A, Svensson PJ. Evaluation of recurrent venous thromboembolism in patients with Factor v Leiden mutation in heterozygous form. Thromb Res. 2012;130(3):467-71.

18. Olie V, Zhu T, Martinez I, Scarabin PY, Emmerich J. Sex-specific risk factors for recurrent venous thromboembolism. Thromb Res. 2012;130(1):16-20.

19. Weingarz L, Schindewolf M, Schwonberg J, Hecking C, Wolf Z, Erbe M, et al. Thrombophilia and risk of VTE recurrence according to the age at the time of first VTE manifestation. Vasa. 2015;44(4):313-23.

20. Franco Moreno AI, Garcia Navarro MJ, Ortiz Sanchez J, Martin Diaz RM, Madronal Cerezo E, de Ancos Aracil CL, et al. A risk score for prediction of recurrence in patients with unprovoked venous thromboembolism (DAMOVES). Eur. 2016;29:59-64.

21. Bruzelius M, Ljungqvist M, Bottai M, Bergendal A, Strawbridge RJ, Holmstrom M, et al. F11 is associated with recurrent VTE in women. A prospective cohort study. Thromb Haemost. 2016;115(2):406-14.

22. Mean M, Limacher A, Stalder O, Angelillo-Scherrer A, Alberio L, Fontana P, et al. Do Factor V Leiden and Prothrombin G20210A Mutations Predict Recurrent Venous Thromboembolism in Older Patients? Am J Med. 2017;130(10):1220.e17-.e22.

23. Limperger V, Kenet G, Kiesau B, Kother M, Schmeiser M, Langer F, et al. Role of prothrombin 19911 A>G polymorphism, blood group and male gender in patients with venous thromboembolism: Results of a German cohort study. J Thromb Thrombolysis. 2020;51(2):494-501.

24. Hodeib H, Youssef A, Allam AA, Selim A, Tawfik MA, Abosamak MF, et al. Genetic risk profiling associated with recurrent unprovoked venous thromboembolism. Genes. 2021;12(6) (no pagination).
